# The prognostic significance of a postoperative systemic inflammatory response in patients with colorectal cancer

**DOI:** 10.1186/s12957-015-0609-3

**Published:** 2015-06-04

**Authors:** Masatsune Shibutani, Kiyoshi Maeda, Hisashi Nagahara, Hiroshi Ohtani, Yasuhito Iseki, Tetsuro Ikeya, Kenji Sugano, Kosei Hirakawa

**Affiliations:** Department of Surgical Oncology, Osaka City University Graduate School of Medicine, 1-4-3 Asahi-machi Abeno–Ku Osaka City Osaka Prefecture, 545-8585 Abeno–ku, Osaka, Japan

**Keywords:** Colorectal cancer, Systemic inflammatory response, Neutrophil-to-lymphocyte ratio, Prognosis

## Abstract

**Background:**

Recently, a preoperative systemic inflammatory response has been reported to be a prognostic factor in patients with colorectal cancer (CRC). However, the prognostic significance of a systemic inflammatory response in the early stage after surgery in patients with CRC is unknown. The aim of this retrospective study was to evaluate the prognostic significance of a postoperative systemic inflammatory response in patients with CRC.

**Methods:**

Two hundred and fifty-four patients who underwent potentially curative surgery for stage II/III CRC were enrolled in this study. Univariate and multivariate analyses were performed to evaluate the relationship between the prognosis and clinicopathological factors, including the neutrophil-to-lymphocyte ratio (NLR) and Glasgow Prognostic Score (GPS), which were measured within two weeks before operation and at the first visit after leaving the hospital.

**Results:**

The overall survival rates were significantly worse in the high preoperative NLR/preoperative GPS/postoperative NLR group. A multivariate analysis indicated that only preoperative GPS, postoperative NLR, and the number of lymph node metastases were independent prognostic factors for a poor survival.

**Conclusions:**

The postoperative NLR is an independent prognostic factor in patients with CRC who underwent potentially curative surgery.

## Background

Colorectal cancer (CRC) is the third leading cause of cancer death worldwide [1]. Although the surgical procedures and chemotherapy have improved, a large number of patients relapse after curative resection, and the mortality from colorectal cancer is still high. Therefore, it is necessary to identify the patients with a high possibility of recurrence, and various biomarkers associated with poor survival have been examined.

Recently, the systemic inflammatory response has been recognized to correlate with the progression of the tumor and the prognosis of various types of cancer, including CRC. The markers of the systemic inflammatory response, such as the neutrophil-to-lymphocyte ratio (NLR) [2–4], serum C-reactive protein (CRP) level [5, 6], and Glasgow prognostic score (GPS) [4, 7, 8] have been reported to be associated with the prognosis in patients with CRC. However, most of these reports investigated the preoperative status, and there have been no reports on the relationship between the systemic inflammatory response in the early stage after surgery and the prognosis after potentially curative resection of CRC. The aim of this retrospective study was to evaluate the prognostic significance of the postoperative systemic inflammatory response in patients with CRC.

## Methods

We retrospectively reviewed a database of 254 patients who underwent potentially curative surgery for stage II/III CRC at the Department of Surgical Oncology of Osaka City University between 2006 and 2011. Curative surgery was defined as the absence of any gross residual tumor tissue in the surgical bed, with a surgical resection margin that was pathologically negative for tumor invasion. Patients who received preoperative therapy or who had either bowel obstruction or perforation due to their primary tumor were excluded from the analysis.

The patient population consisted of 139 males and 115 females, with a median age of 60 years (range, 26 to 86). One hundred and thirty-one patients had tumors located in the colon, and 123 had tumors located in the rectum. One hundred and seventy-eight patients received monotherapy using an oral pro-drug based on 5-FU, such as capecitabine, while 30 patients received combination therapy with 5-FU and oxaliplatin, such as 5-fluorouracil/leucovorin plus oxaliplatin (FOLFOX) or capecitabine plus oxaliplatin (CapeOX) (Table 1).

**Table 1:** The patient characteristics

Gender	
Male	139
Female	115
Age (years)	
Median (range)	66 (26–86)
Location of primary tumor	
Colon	131
Rectum	123
Tumor depth	
T1-3	176
T4	77
Histological type	
Well, moderately	234
Poorly, mucinous	19
Lymphatic involvement	
Negative	47
Positive	184
Venous involvement	
Negative	170
Positive	68
Number of lymph node metastases	
0	85
1–3	116
≥4	53
Stage	
II	85
III	169
Regimen of chemotherapy	
Oral 5-FU monotherapy	178
CapeOX	19
FOLFOX	11
None	46
Median value of indicators of the preoperative systemic inflammatory response (range)	
NLR	2.26 (0.87–10.24)
CRP (mg/dl)	0.11 (0.01–13.99)
Preoperative serum albumin level (g/dl)	
Median (range)	4.1 (2.6–4.8)
Median value of indicators of the postoperative systemic inflammatory response (range)	
NLR	1.82 (0.18–10.11)
CRP (mg/dl)	0.09 (0.01–17.09)
Postoperative serum albumin level (g/dl)	
Median (range)	4.0 (3.0–4.7)
The number of days from operation until the first visit after leaving the hospital	
Median (interquartile range)	29 (23–36)

*5-FU* 5-fluorouracil, *CapeOX* capecitabine plus oxaliplatin, *FOLFOX* 5-fluorouracil/leucovorin plus oxaliplatin, *NLR* neutrophil-to-lymphocyte ratio, *CRP* C-reactive protein

**Fig. 1:**
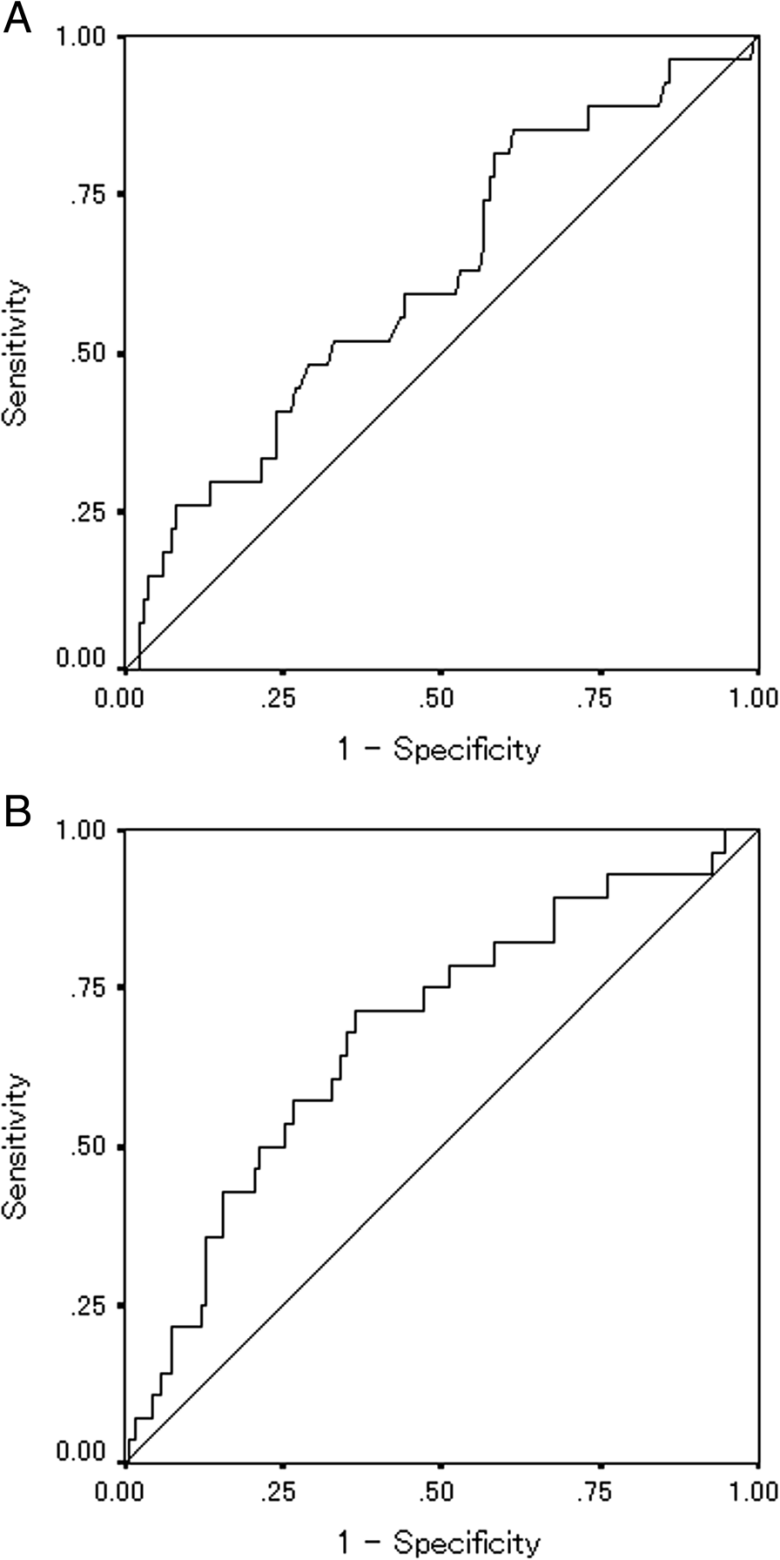
**a** Receiver-operating characteristic-curve analysis of the preoperative NLR. Area *under* the curve = 0.618, 95 % confidence interval = 0.502–0.735, *p* = 0.053. **b** Receiver-operating characteristic-curve analysis of the postoperative NLR. Area *under* the curve = 0.680, 95 % confidence interval = 0.573–0.787, *p* = 0.002

The postoperative systemic inflammatory response was measured at the first visit after leaving the hospital. The date of the first visit was set to occur two to three weeks after the patient left the hospital. The median (interquartile range) period from the operation until the first visit after leaving the hospital was 29 (23–36) days. The NLR was calculated from a blood sample by dividing the absolute neutrophil count by the absolute lymphocyte count. According to the receiver-operating characteristic (ROC) curve, we set 2.5 as the cut-off value for the preoperative NLR (the sensitivity was 51.9 % and the specificity was 64.2 %) (Fig. 1a) and classified the patients into high preoperative NLR (≥2.5) and low preoperative NLR (<2.5) groups. Moreover, according to the ROC curve, we also set 3.0 as the cut-off value for the postoperative NLR (the sensitivity was 35.7 % and the specificity was 87.3 %) (Fig. 1b) and classified the patients into high postoperative NLR (≥3.0) and low-postoperative NLR (<3.0) groups.

We defined the GPS according to the previous reports as follows [9]: the GPS consists of the combination of an elevated CRP (≥1 mg/dl) and hypoalbuminemia (<3.5 g/dl). Patients with both abnormalities were allocated a GPS of 2. Patients with only one of these abnormalities were allocated a GPS of 1. Patients with normal values for both were allocated a GPS of 0. The patients with a GPS of 1 or 2 were classified into the high GPS group, and those with a GPS of 0 were classified into the low-GPS group.

We then examined the correlations between the clinicopathological parameters, including the postoperative NLR/GPS and the prognosis for survival. All patients were followed up regularly with physical and blood examinations and mandatory screening using colonoscopy and computed tomography until May 2014 or death. Among the total 254 patients, 86 developed recurrent disease and 42 patients died.

The resected specimens were pathologically classified according to the seventh edition of the Union for International Cancer Control TNM classification of malignant tumors [10]. The significance of the correlations between the systemic inflammatory response and the clinicopathological characteristics was analyzed by the χ^2^ test, Fisher’s exact test, and *t*-test. The duration of survival was calculated according to the Kaplan-Meier method. Differences in the survival curves were assessed with the log-rank test. A multivariate analysis was performed according to the Cox proportional hazards model. All statistical analyses were conducted using the SPSS software package for Windows (SPSS Japan, Tokyo, Japan). Statistical significance was set at a value of *p* <0.05.

## Results

The preoperative/postoperative indicators of a systemic inflammatory response are shown in Table 1. The distribution of patients based on the indicators of a systemic inflammatory response is shown in Table 2.

**Table 2:** The distribution of patients based on the indicators of the postoperative systemic inflammatory response

	Preoperation	Postoperation
NLR		
Low	99 (61.5 %)	183 (84.3 %)
High	62 (38.5 %)	34 (15.7 %)
GPS		
0	174 (77.7 %)	159 (77.6 %)
1	44 (19.6 %)	39 (19.0 %)
2	6 (2.7 %)	7 (3.4 %)

*NLR* neutrophil-to-lymphocyte ratio, *GPS* Glasgow prognostic score

**Fig. 2:**
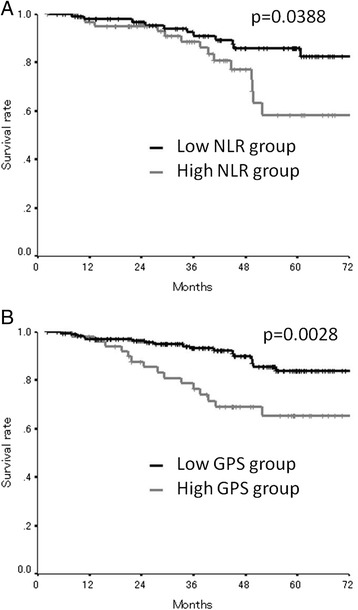
**a** The overall survival according to the preoperative NLR. The overall survival rates were significantly worse in the high preoperative NLR group (*p* = 0.0388). **b** The overall survival according to the preoperative GPS. The overall survival rates were significantly worse in the high preoperative GPS group (*p* = 0.0028)

**Fig. 3:**
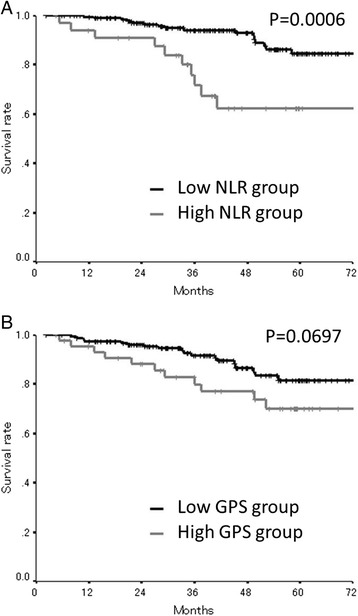
**a** The overall survival according to the postoperative NLR. The overall survival rates were significantly worse in the high postoperative NLR group (*p* = 0.0006). **b** The overall survival according to the postoperative GPS. There was no relationship between the postoperative GPS and mortality

As for the preoperative inflammatory status, an assessment of the prognosis showed that the overall survival rates were significantly worse in the high preoperative NLR/GPS group (NLR, *p* = 0.0388; GPS, *p* = 0.0028) (Fig. 2). Moreover, as for the postoperative inflammatory status, the overall survival rates were significantly worse in the high postoperative NLR group (*p* = 0.0006), while there was no relationship between the postoperative GPS and mortality (Fig. 3). The postoperative NLR had a significant relationship with the amount of blood loss during the operation and the length of the operation and tended to correlated with gender, while there was no relationship between the postoperative NLR and other factors including preoperative NLR (Table 3). The postoperative GPS had a significant relationship with lymphatic involvement, the number of lymph node metastasis, the preoperative CA19-9 level, and the preoperative GPS (Table 3). With regard to the relationships between the postoperative systemic inflammatory response and the sub-classification of the postoperative infectious complications, neither NLR nor GPS showed a significant relationship with the sub-classification of the postoperative infectious complications (Table 4).

**Table 3:** The correlation between the postoperative systemic inflammatory response and the clinicopathological factors

	Postoperative NLR			Postoperative GPS		
	<3	≥3	*p* value	0	1,2	*p* value
Age (years)						
<70	120	19		102	28	
≥70	63	15	0.331	57	18	0.729
Gender						
Male	97	24		84	27	
Female	86	10	0.063	75	19	0.506
Location						
Colon	93	19		86	22	
Rectum	90	15	0.709	73	24	0.504
Tumor depth						
T1-3	133	22		112	33	
T4	49	12	0.406	47	13	1.000
Histological type						
Well, moderately	170	31		147	43	
Poorly, mucinous	12	3	0.711	12	2	0.739
Lymphatic involvement						
Negative	39	5		35	3	
Positive	124	27	0.363	112	37	0.026
Venous involvement						
Negative	123	24		109	35	
Positive	46	9	1.000	41	8	0.321
Number of lymph node metastases						
0	75	8		60	7	
1–3	71	19		64	30	
≥4	37	7	0.116	35	9	0.005
Preoperative CEA (>5 ng/ml)						
Negative	129	25		116	29	
Positive	38	6	0.816	35	8	1.000
Preoperative CA19-9 (>37 U/ml)						
Negative	158	28		145	33	
Positive	5	2	0.298	3	4	0.031
Adjuvant chemotherapy						
No	40	4		35	8	
Yes	143	30	0.246	124	28	0.545
Length of operation (min)						
Median (range)	199 (79–430)	230 (84–687)	0.010	203 (79–687)	206 (110–372)	0.681
Blood loss (ml)						
Median (range)	80 (5–1785)	220 (10–2700)	<0.001	80 (5–2700)	90 (10–1880)	0.495
Postoperative infectious complication						
No	137	25		121	34	
Yes	46	9	0.833	38	12	0.846
Preoperative NLR						
<2.5	70	12				
≥2.5	45	8	1.000			
Preoperative GPS						
0				131	21	
1,2				24	20	<0.001

*NLR* neutrophil-to-lymphocyte ratio, *GPS* Glasgow prognostic score, *CEA* carcinoembryonic antigen, *CA19-9* carbohydrate antigen 19-9

**Table 4:** The correlation between the postoperative systemic inflammatory response and the sub-classification of the postoperative infectious complications

	Postoperative NLR			Postoperative GPS		
	<3	≥3	*p* value	0	1,2	*p* value
Criteria according to Clavien-Dindo classification						
Without complication, grade I	131	21		113	33	
Grade ≥II	50	13	0.223	45	13	1.000
Wound infection						
No	168	33		150	41	
Yes	15	1	0.477	9	5	0.315
Anastomotic leakage						
No	171	31		149	42	
Yes	12	3	0.710	10	4	0.521
Abdominal abscess						
No	176	33		154	45	
Yes	7	1	1.000	5	1	1.000
Enterocolitis						
No	176	33		153	46	
Yes	7	1	1.000	6	0	0.341
Pneumonia						
No	183	33		158	46	
Yes	0	1	0.157	1	0	1.000
Urinary tract infection						
No	181	33		157	45	
Yes	2	1	0.402	2	1	0.535
Duodenal perforation						
No	183	33		158	46	
Yes	0	1	0.157	1	0	1.000

*NLR* neutrophil-to-lymphocyte ratio, *GPS* Glasgow prognostic score

The correlations between the overall survival and various clinicopathological factors are shown in Table 5. According to a univariate analysis, the overall survival had significant relationships with the postoperative NLR, the preoperative NLR, the preoperative GPS, age, the tumor depth, histological type, venous involvement, and the number of lymph node metastases. However, a multivariate analysis indicated that only the preoperative GPS, the postoperative NLR, and the number of lymph node metastases were independent risk factors for mortality.

**Table 5:** The correlations between the overall survival and various clinicopathological factors

	Univariate analysis			Multivariate analysis		
	Hazard ratio	95 % CI	*p* value	Hazard ratio	95 % CI	*p* value
Age (>70 years)	2.113	1.142–3.911	0.017	0.912	0.204–4.083	0.904
Gender (Male)	0.684	0.361–1.295	0.243			
Location of primary tumor (Colon)	0.749	0.404-1.389	0.360			
Tumor depth (T4)	1.863	1.007–3.448	0.048	4.592	0.896–23.544	0.068
Histological type (Poorly, mucinous)	3.449	1.582–7.518	0.002	0	0	0.988
Lymphatic involvement (Positive)	2.744	0.839–8.979	0.095			
Venous involvement (Positive)	2.102	1.080–4.093	0.029	0.350	0.068–1.800	0.209
Number of lymph node metastases	2.924	1.816–4.707	<0.001	14.677	2.571–83.779	0.003
Preoperative CEA (>5 ng/ml)	1.939	0.875–4.299	0.103			
Preoperative CA19-9 (>37 U/ml)	1.298	0.176–9.586	0.798			
Adjuvant chemotherapy (Yes)	0.332	0.080–1.384	0.130			
Chemotherapy regimen (with oxaliplatin)	0.726	0.216–2.433	0.603			
Postoperative NLR (>3.0)	3.597	1.643–7.875	0.001	15.713	1.590–155.227	0.018
Postoperative GPS (≥1)	1.982	0.933–4.208	0.075			
Preoperative NLR (>2.5)	2.204	1.023–4.750	0.044	6.599	0.928–46.914	0.059
Preoperative GPS (≥1)	2.723	1.372–5.404	0.004	7.238	1.180–44.415	0.032

*CEA* carcinoembryonic antigen, *CA19-9* carbohydrate antigen 19-9, *NLR* neutrophil-to-lymphocyte ratio, *CRP* C-reactive protein, *GPS* Glasgow prognostic score

**Fig. 4:**
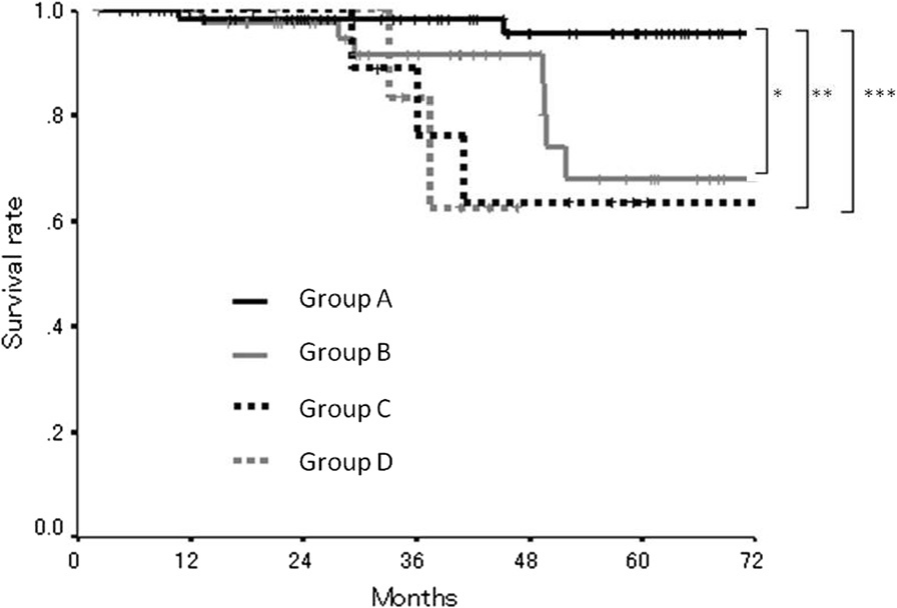
The overall survival subdivided according to the preoperative and postoperative NLR. The patients in group A exhibited a better prognosis compared to the other groups (*p = 0.0124; **p = 0.0202; ***p = 0.0031)

We categorized the patients into four groups according to the combination of their preoperative and postoperative NLR. Patients with the low preoperative and postoperative NLR categorized into group A. Patients with the low preoperative NLR and the high postoperative NLR were categorized into group B. Patients with the high preoperative NLR and the low-postoperative NLR were categorized into group C. Patients with the high preoperative and postoperative NLR categorized into group D. The patients in group A exhibited a better prognosis compared to the other groups (AvsB, *p* = 0.0124; AvsC, *p* = 0.0202; AvsD, *p* = 0.0031), while there was no significant difference between groups B, C, and D with regard to survival (Fig. 4).

## Discussion

In this study, we investigated the correlations between the high postoperative NLR and poor survival in patients with colorectal cancer who underwent potentially curative surgery. When considering the prognosis of patients with malignant tumors, the TNM-classification criteria [10], which are factors related to the tumor and accurately reflect the prognosis, have been widely used. Recently, the prognostic significance of the factors related to the host based on the systemic inflammatory response, such as the NLR, CRP, and GPS in patients with CRC, has been reported [2–8]. However, most of the previous reports focused on the preoperative status, and there have been only a few reports which focused on the prognostic significance of the postoperative systemic inflammatory response. To the best of our knowledge, this is the first study assessing the prognostic significance of the systemic inflammatory response in the early stage after surgery.

Neutrophils play a key role in tumor progression, producing a number of ligands that induce tumor cell proliferation and invasion, and promoting tumor vascularization by releasing proangiogenic chemokines and other factors [11, 12]. As the main cause of recurrence after potentially curative operation may be the growth of micrometastases which had been established prior to resection [13], and because the continuous systemic inflammatory response creates a favorable environment for micrometastatic growth, a persistently elevated level of neutrophils after surgery is considered to correlate with the development of recurrence. In contrast, lymphocytes, which play an important role in anti-tumor immunity, are a factor related to the immune system of the host [14]. The absolute lymphocyte count is assumed to reflect the degree of responsiveness of a cancer patient’s whole immune system [15]. Therefore, a decrease of lymphocytes is considered to correlate with recurrence. Taken together, a persistently high NLR after surgery means the continuation of an environment that is favorable for recurrence. Thus, the postoperative status, as well as the preoperative status of the host, is important when considering the prognosis.

The mechanism of the persistent activation of the systemic inflammatory response after surgery remains unclear. In this study, a high postoperative NLR was significantly correlated with the amount of blood loss during the operation and the length of the operation. These results suggested that a high postoperative NLR might be associated with higher surgical stress. However, we could not conclude that the main cause of the persistent elevation of the systemic inflammatory response after the operation was surgical stress itself, because other than the parameters of blood loss during the operation and the length of the operation, there are no useful markers for evaluating the degree of surgical stress, and the markers on their own were not sufficient to perform an evaluation. On the other hand, the postoperative NLR had no association with the factors related to the tumor, although the preoperative NLR was previously reported to correlate with several factors related to the tumor [2]. Moreover, the postoperative NLR had no relationship with the presence of postoperative infectious complications, even when performing the additional analyses regarding the degree and type of postoperative infectious complications. There were some patients with normal inflammatory marker levels at the first visit after leaving the hospital who developed postoperative infectious complications, while some patients with high postoperative systemic inflammatory marker levels were discharged without postoperative complications. The postoperative infectious complications may not be the main cause of the high postoperative systemic inflammatory response at the first visit after leaving the hospital. Aside from surgical stress and the postoperative infectious complications, the response of the host to the micrometastatic lesion has been reported to cause a persistently high postoperative systemic inflammatory response [16]. However, it is questionable whether the response to the micrometastatic lesion and the response to the primary tumor are equivalent.

Our results were in line with a study by Guthrie et al., which reported that the persistent elevation of the systemic inflammatory response after surgery was correlated with poor survival [16]. However, we obtained different results in relation to the superiority of the postoperative inflammatory markers. We found postoperative NLR to be superior to the postoperative GPS, while Guthrie et al. reported the opposite [16]. Moreover, the timing of the valuation of the postoperative inflammatory response differed between this study and the previous report. In this study the postoperative inflammatory response was evaluated in the early stage after operation (approximately 1–2 months after surgery, when we decided the regimen of adjuvant chemotherapy), while in the previous report, the inflammatory response was evaluated at 3–6 months after surgery [16].

There are some limitations associated with this study. First, we evaluated a relatively small number of patients. Second, the criteria for the first visit after leaving the hospital were not uniform because this study was a retrospective study. Third, the appropriate timing for the evaluation of the postoperative systemic inflammatory response to predict the survival was unknown. Fourth, the mechanism of the persistent elevation of the postoperative inflammatory response remains unclear. A large, prospective study should therefore be performed to confirm our findings.

## Conclusions

In this study, the postoperative NLR was demonstrated to correlate with a poor survival as well as the preoperative NLR and the postoperative NLR were investigated to be an independent prognostic factor for poor survival. Therefore, not only the preoperative status of the host, but also the postoperative status of the host, is important when considering the prognosis.

## Abbreviations

CA19-9: carbohydrate antigen 19–9
CEA: carcinoembryonic antigen
CapeOX: capecitabine plus oxaliplatin
CRC: colorectal cancer
CRP: C-reactive protein
FOLFOX: 5-fluorouracil/leucovorin plus oxaliplatin
GPS: Glasgow prognostic score
NLR: neutrophil-to-lymphocyte ratio
ROC: receiver-operating characteristic
